# Modelling human Puumala hantavirus infection in relation to bank vole abundance and masting intensity in the Netherlands

**DOI:** 10.1080/20008686.2017.1287986

**Published:** 2017-03-24

**Authors:** Arno Swart, Dick L. Bekker, Miriam Maas, Ankje de Vries, Roan Pijnacker, Chantal B. E. M. Reusken, Joke W. B. van der Giessen

**Affiliations:** ^a^ Centre for Infectious Disease Control, National Institute for Public Health and the Environment, Bilthoven, The Netherlands; ^b^ Dutch Mammal Society, Nijmegen, the Netherlands; ^c^ Detail 2.0 – Faunistical Research, Groningen, the Netherlands; ^d^ Department of Viroscience, Erasmus University Medical Centre, Rotterdam, the Netherlands

**Keywords:** Puumala, human cases, prediction, environment, climate

## Abstract

This paper deals with modelling the relationship between human Puumala hantavirus (PUUV) infection, the abundance and prevalence of infection of the host (the bank vole), mast, and temperature. These data were used to build and parametrise generalised regression models, and parametrise them using datasets on these factors pertaining to the Netherlands. The performance of the models was assessed by considering their predictive power. Models including mast and monthly temperature performed well, and showed that mast intensity influences vole abundance and hence human exposure for the following year. Thus, the model can aid in forecasting of human illness cases, since (1) mast intensity influences the vole abundance and hence human exposure for the following year and (2) monitoring of mast is much more feasible than determining bank vole abundance.

## Introduction

European hantaviruses are a concern for public health, since they can cause influenza-like infection in humans, and may also lead to the more serious haemorrhagic fever with renal syndrome. In north and west Europe, the prevailing disease is nephropathia epidemica (NE), which is relatively mild. Each hantavirus species is typically thought to be associated with one main specific mammalian host. In this paper we consider Puumala hantavirus (PUUV), which is known to cause human infections, and is associated with the bank vole (*Myodes glareolus*). PUUV is excreted by voles in faeces and urine, which may be aerosolised from the environment, become airborne, and subsequently infect humans. Human infection is therefore, amongst other things, dependent on the abundance and prevalence of infection of bank voles. Furthermore, climate (temperature, moisture) will be important for survival of the virus in the environment, and will influence rodent survival. In [1] it is shown that PUUV hantavirus may survive up to 11 days at room temperature, and up to 18 days at 4ºC, indicating that human infection from the environment is a feasible scenario. Finally, human behaviour plays a role in human exposure. For example, there may be more recreation in forests, the preferred habitat of bank voles, in the summer months, when temperatures are inviting.[2] Furthermore, human activities like cleaning a dusty stable are potentially risky, since this may disturb settled contaminated aerosols, releasing them into the air, making them available for inhalation.

The population dynamics of bank voles differs greatly between the northern boreal and western temperate regions of Europe. In northern regions, bank vole populations are driven by predator–prey dynamics,[3] yielding seasonal and multi-annual patterns in human NE infections.[4] In contrast, in western European regions bank vole population dynamics are to a large extent determined by the availability of staple food: nuts and acorns, collectively known as mast.[5–7] It is an intriguing hypothesis that the cyclic nature of human outbreaks may be connected to peak mast years, which also occur in multi-annual cycles.[8] In [9] univariate analyses were performed, relating human PUUV infection to mast and climatic factors. It was found that mast in the previous year is an important predictor for human outbreaks, as well as warmer autumns the year before, warmer summers two years before, but also colder and moister summers three years before outbreaks. In [10] this result is further strengthened using generalised linear models. In particular, high summer and autumn temperatures respectively one and two years before an outbreak were found to be significant predictors of human PUUV infection.

For public health interventions aiming at preventing human PUUV infection, it is desirable to have a system in place that can predict risk of PUUV infection. To this end, it is possible to monitor the bank vole population, but this is not feasible on a large (country-wide) scale, especially in countries like the Netherlands where voles have a very patchy distribution due to fragmented land attributes. A model that can predict risk of exposure to PUUV based on parameters that are more easily monitored is desired.

A diverse body of literature exists on modelling infections of humans and voles, bank vole populations, mast, and their inter-relationships. A spatial analysis of determinants of human hantavirus infections was performed.[11] Several drivers of enhanced risk were identified, e.g. connectivity of forests, vegetation activity, low soil water content, mild summers, cold winters, proportion of built-up areas in forest ecotones, and a lower minimum temperature in winter. Published models typically are limited to certain aspects (voles, mast, climate, human infection) of the causal chain leading to human infection. We aimed to make a synthesis, and connect the aspects of (1) vole abundance; (2) vole infection prevalence; (3) mast; and (4) human cases, by constructing predictive models. The model was parametrised based on several datasets pertaining to the Netherlands, including seroprevalence and abundance data of a localised bank vole population that was monitored for seven years.[12] It was able to predict the rate of new human cases from infected vole prevalence and vole abundance, or from mast data. Thus, masting, which is simpler to measure in the field, was predictive for human cases. Even more, it was the mast score of the previous year which is of relevance for the bank vole population densities at the current year, making the masting volume also a timely indicator. The vole infection status was a significant factor, which however did not contribute greatly to the model predictions. Thus, though it has added value to know the infection prevalence in the vole population, this knowledge is not required in the model for risk predictions.

## Material and methods

### Mast

Information on masting was obtained from several sources (Table 1). Each reference has its own measure of mast intensity, depending on the method used. We considered oak (*Quercus robur*) and beech (*Fagus sylvatica*), the main food sources of the bank vole, in the Netherlands, between 2006 and 2014. To have a uniform dataset, all data were transformed to a common five-point scale: (0) no mast, (1) very low mast production, (2) low mast production, (3) normal mast production, (4) abundant mast production, (5) very abundant mast production. The references in the table point to the conversion methods used.

**Table 1.** Sources of mast data.

Source	Years	Method	Reference
Vereniging Wildbeheer Veluwe	2006–2013	Weight of mast	Unpublished
Alterra	2006–2013	Weight of mast	[13]
IPC	2003–2014	Score	[14]
G.J. Spek	1987–2014	Weight of mast	Personal communication
Vilmar Dijkstra	2007–2008, 2013–2014	Count of mast	Personal communication
NAK tuinbouw	2002–2014	Score	Personal communication

### Bank voles

Bank voles were captured in the Dutch region Twente, where the majority of human PUUV infections occur in the Netherlands, as described in [12]. Briefly, twice a year, in July and October, rodents were captured. Seven trapping sites, consisting of 100 traps in total, were set in the area, and rodents were collected at four occasions: day one in the evening, day two in the morning and in the evening and day three in the morning. The number of bank voles captured followed a complicated pattern over the four capture occasions. A study of this dynamics is outside of the scope of this study, and we simply averaged the four values. This number is considered as a measure proportional to the abundance, but will simply be denoted by ‘the abundance’ from here on. Though other rodent and insectivore species were also captured, for this study only the bank vole was considered since it is the main host for PUUV.

For October 2007, the original data on bank vole captures were missing, but data pertaining to the serological testing of the voles were still available. These data on the numbers of voles tested were used as a surrogate, although this may give an under-estimation since not all voles captured were tested because some, e.g. pregnant voles, were released.

### Vole infection status

The captured voles were tested for the presence of PUUV antibodies by a rapid immunochromatography test. Details of the procedure are supplied in [12]. By dividing the number of positive voles by the total number of voles tested, we obtained the prevalence of PUUV infection. This prevalence estimate was subsequently applied to the total number of captured voles, to obtain the abundance of infected voles.

### Human cases

PUUV infections have been notifiable in the Netherlands since December 2008 and voluntary laboratory surveillance has been in place since 1989. Detailed epidemiological reports of hantavirus, presumably PUUV, cases were provided by the Municipal Public Health Services (GGD) to the National Institute for Public Health and the Environment (RIVM).[15] For our purposes, only cases that acquired their infection within the Netherlands were included. If multiple cases were likely infected by the same source within a two-week time period, only one case was included. Cases were assigned to a year and month based on their day of illness onset and if unknown, date of diagnosis.

### Temperature

Monthly temperatures were downloaded from the Royal Dutch Meteorological Institute (KNMI) at http://www.knmi.nl/nederland-nu/klimatologie/maandgegevens. Temperatures were averaged over all measuring stations, and further averaged over each month per year.

### Model

The modelling components comprise of several steps. First, the bank vole abundance was inferred from masting data. Second, human cases were inferred from the abundance of voles, including and excluding vole infection status. Finally, human cases were predicted directly from the mast data. For the models that aim to predict human cases, temperature was optionally included.

#### Modelling vole abundance

A Poisson regression model for the abundance was built, with 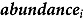 the 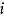
th data point for the abundance,

(1) 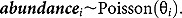

A Poisson model is chosen since the data are in the form of count data. The rate is modelled as:

(2) 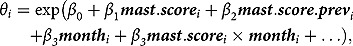

where the ellipsis indicate all possible interactions, which are not written fully. The variable 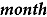 is either 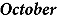 or 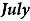, all other covariates are numeric. The variables 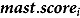 and 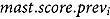 indicate the mast score for the current and previous year. A backward and forward selection based on AIC is used for model selection. The fit of the final model is determined by reading off the chi-squared distribution at the residual deviance of the model fit, with the appropriate degrees of freedom. It was found that the model fit was poor, often indicative of over-dispersion, and subsequently a negative binomial model was fitted to accommodate the excess variance [16]:

(3) 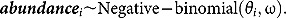

Here, 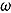 is the over-dispersion parameter, and 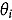 the mean, modelled as before.

#### Modelling human cases from vole abundance

**Figure 1.**
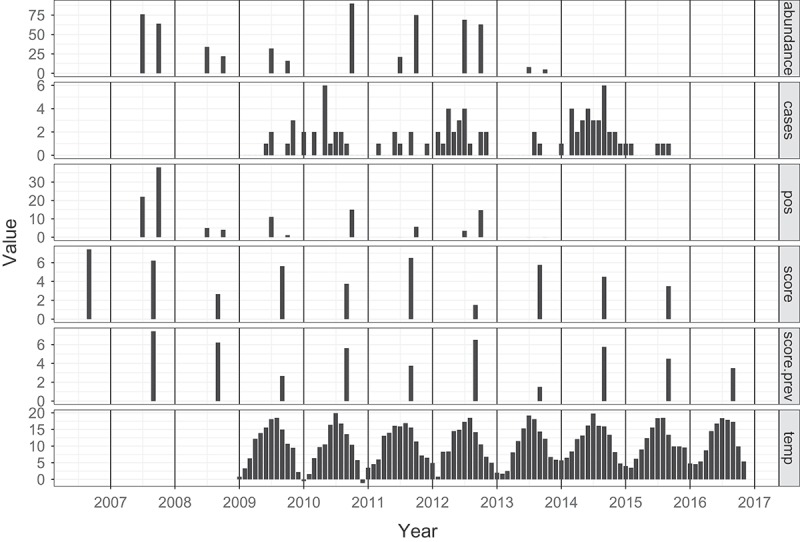
The panels show (1) vole abundance, (2) monthly averaged human PUUV cases, (3) the abundance of PUUV positive voles, (4) the mast score, (5) the mast score of the previous year, and (6) monthly maximum temperature, the Netherlands 2006–2016.

Modelling the number of human cases as a function of positive voles is more complicated. The explanatory data are irregularly spaced estimates of positive voles (Figure 1, panel 3), with monthly human case data (panel 2) as dependent variable. The months since January 2008 are indexed by 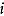, and it is assumed that a case in month 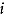 can be partly explained by the infected vole abundance in the first preceding vole observation. As a second explanatory variable the maximum daily temperature is considered (panel 6), averaged over months. The cases 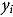 are assumed to be Poisson distributed:

(4) 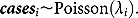

**Figure 2.**
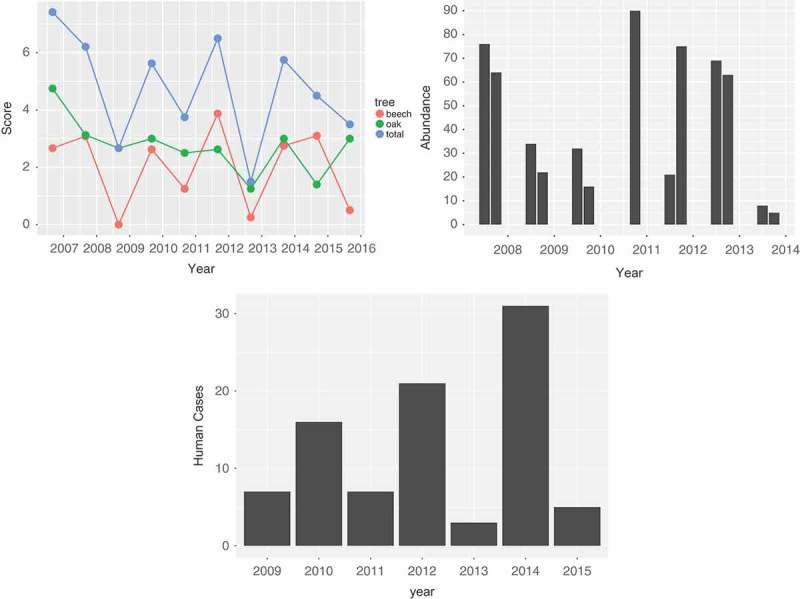
(a) Mast score by year for the Netherlands, 2007–2015. The green line gives the mast score for oak, the red line the score for beech. The blue line is the sum, which we use in the model. (b) Vole abundance in Twente, the Netherlands, for June and October 2008–2014. (c) The total number of human PUUV cases per year, the Netherlands 2009–2015.

A model with fixed 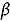 coefficients would have equal weight for each month, irrespective of the time passed since the last observation. This is not realistic, and is remedied by weighing the coefficient with the time passed to the last observation. Let 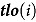 (time of last observation) be the last time, before month 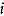, that we have an observation. Thus 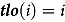 at a month at which there was a vole observation. Denote the vole abundance in month 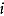 by 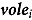 and the abundance of positive voles by 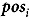. Then, the last observation may be looked up by considering 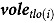. The older the available information, the less it should contribute, which is modelled by weighing the data exponentially:

(5) 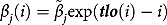

Finally, 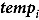 is the temperature in month 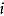. The rate is modelled as:

(6) 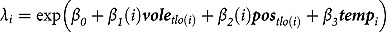

Varying coefficients were implemented by actually having fixed coefficients, but setting interactions of the form 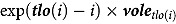, which is equivalent to the above. Backward and forward model selection was performed, and a test for goodness of fit as described above. The years for which the model is fit were limited to before 2014, since vole abundance data was not available for 2014 onward.

#### Modelling human cases from mast data

There is an important advantage in directly modelling human cases from mast data, as it allows for prediction of the next year. We built a model as in the previous section:

(7) 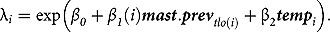

Here, 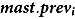 indicates the mast of last year in month 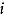. Since the exact month of mast data collection is often not known, the month has been set to September. Therefore, 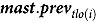 refers to the mast of September the year before.

## Results

### Mast

Figure 2(a) gives the result of the mast determination. Since the exact time of mast determination is unknown or varies, we place the data at September of each year; the assumed month of expected maximum mast volume. Furthermore, the mast score is included in Figure 1, a figure combining all relevant data for the model in one figure, with a common x-axis for time.

### Bank voles

The number of bank voles that were captured, averaged over the trapping events, is given in Figure 2(b). The data for June 2010 were removed, since that year an unusually low number of bank voles were trapped, which could be attributed to a recent mowing of the grass at the road side, near the traps. In general, in Twente, numbers varied between one and 90 with a mean of 30. Abundance in October (mean 40) was somewhat lower than in June (mean 48), and the years 2007, 2010, 2011 and 2012 seemed to have elevated abundance.

### Prevalences

The third panel of Figure 1 gives the abundance of PUUV positive bank voles. A correlation between abundance and prevalence may be postulated, but the *p*-value of the intercept of a linear model relating prevalence to abundance is 0.14, and thus there is no evidence for such a correlation.

### Human cases

From December 2008 to November 2015, 107 PUUV hantavirus cases were reported in the Netherlands, of which 69% (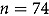) were male. Cases were excluded when they did not contract PUUV in the Netherlands or the country of infection was unknown (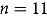) or they contracted the infection at the same location within a short time period as another case (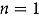). This resulted in 95 cases for these analyses. Date of illness onset was available for 93 cases and two cases were assigned to a year and month based on date of diagnosis. During 2009–2014, the annual number of human PUUV cases has fluctuated between 4 and 37 (see Figure 2(c)).

### Temperature

Maximum monthly temperature data are shown in the bottom panel of Figure 1. It shows that the timing of the cases is mainly determined by the temperature. However, Figure 2(c) also shows a varying pattern of human infection over the years that cannot be explained by the temperature, but which does seem to mimic the mast pattern (offset by one year).

### Model

#### Predicting voles from mast

After model selection, the negative binomial model was found to fit the data satisfactorily (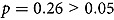). Only the intercept (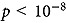) and mast of the previous year (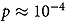) are significant. The final model is

(8) 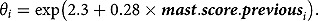

**Figure 3.**
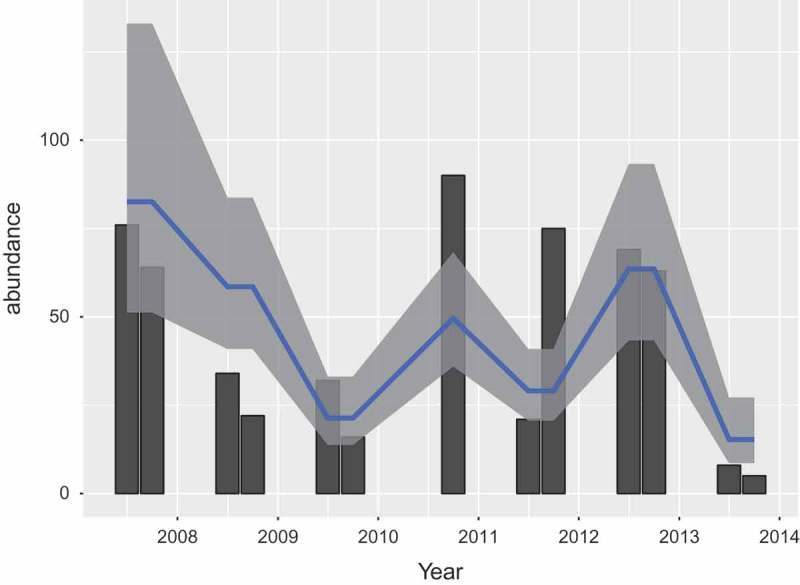
Prediction of vole abundance from mast score. The measured mast score is indicated by the bars, the prediction and 95% confidence interval by the blue line and grey band.

The over-dispersion coefficient is 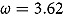. Predictions generated by this model are displayed in Figure 3. Note how the model does not distinguish between months. The interpretation of the model is that there is a baseline of 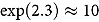 voles captured. Each point of mast score of the previous year increases the number of expected captured voles by a factor 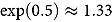, or 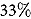.

#### Predicting human cases from vole abundance

For human cases, again the negative binomial model fitted best. After model selection, there is no evidence for lack of fit (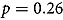). The model contains the last measured abundance of positive voles 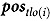 at 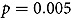. The decaying coefficient 
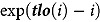
 is slightly over the significance level at 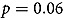, but the interaction between voles and time to last observation is significant at 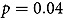. Finally, the temperature 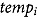 is just significant 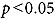. The final model is:

(9) 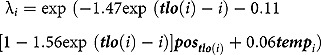

**Figure 4.**
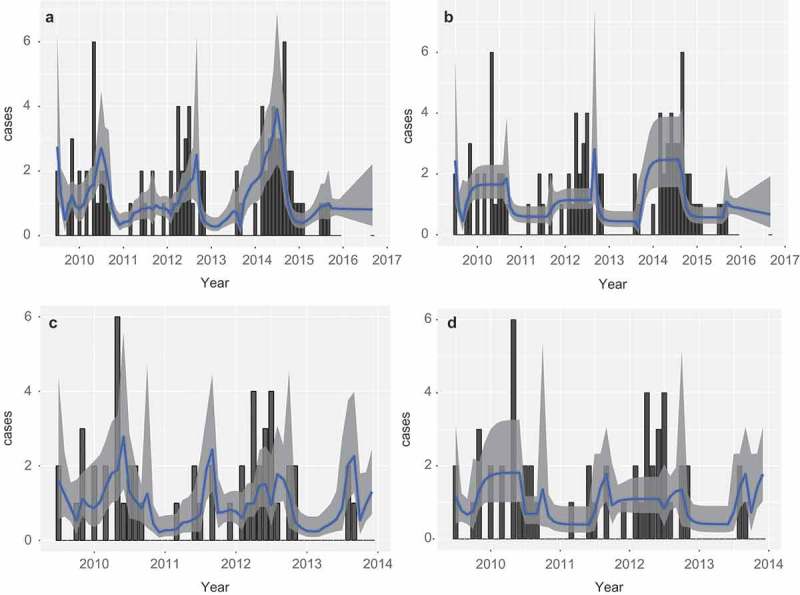
Human cases (bars) and model prediction of the monthly rate of infection with 95% prediction intervals. Panels (a)and (b) are based on mast data, including and excluding monthly temperature, respectively. Panels (c) and (d) are based on based on infected vole abundance, including and excluding monthly temperature, respectively.

Since the temperature was borderline significant, we predicted cases with and without temperature. As Figure 4(c) shows, when temperature is included, the predicted rate of generation of cases per month is in good agreement with the observed number of cases.

Interestingly, model predictions without the influence of temperature, as in Figure 4(d), reproduce the overall pattern over the years, but the timing within the year does not match the cases. It seems that temperature is important for the spread of the cases over the year, not the actual number of cases. This suggests that temperature is mainly an exposure covariate, while masting is a hazard covariate.

#### Predicting human cases from mast data

After model selection, there is no evidence for lack of fit (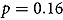). The mast score of the previous year was significant, at 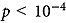. The interaction with time to last observation was significant at 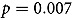, as was the time to last observation itself (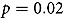). For temperature (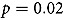), the model was again built with temperature both included and excluded. The final model becomes:

(10) 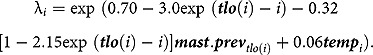

Figures 4(a) and 4(b) show the result for the model prediction including and excluding temperature.

#### Comparison of models

**Figure 5.**
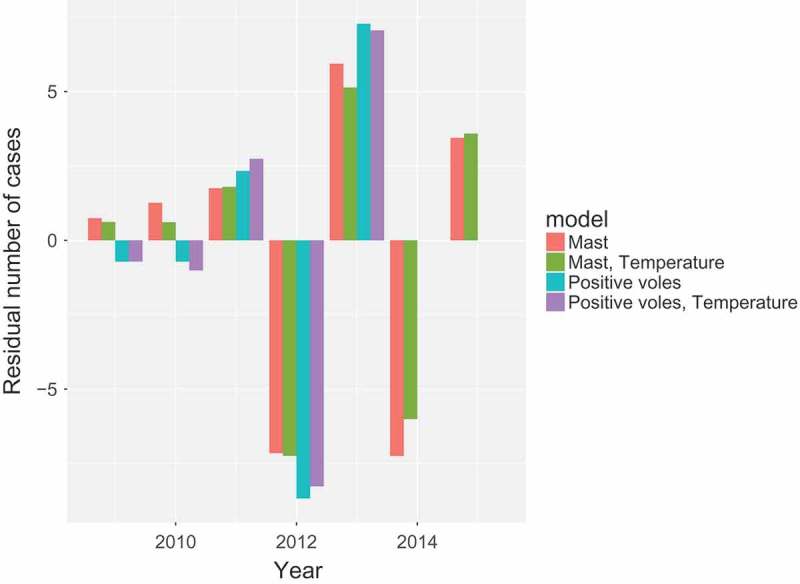
Residuals between model prediction and actual human cases, aggregated by year. Coloured bars indicate the different models. Models depending on vole abundance can only predict up to year 2013 due to data unavailability.

The models discussed in the previous sections are compared in Figure 5. This is done by aggregating both the predicted rate of new cases and the actual number of cases over years. The residual is plotted, which is the difference between the two. Very little difference between the models is found, the mast works as well as abundance of positive voles, and temperature only defines the within-year distribution of cases more sharply.

#### Predictive power

**Figure 6.**
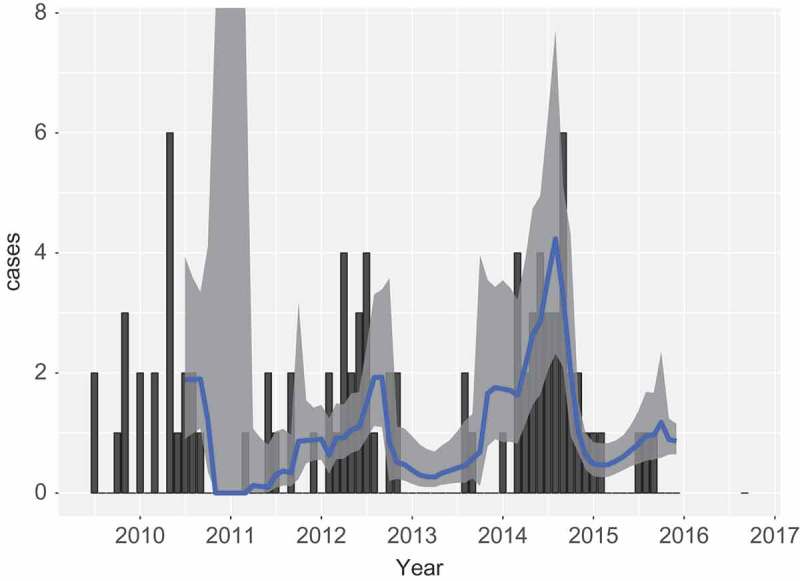
Human cases (bars) and model prediction of the monthly rate of infection with 95% prediction intervals, based on mast data. Only data from before the point of prediction is included in the model. The model takes monthly temperature into account.

The predictions as performed thus far are unfair in the sense that all data are used for the prediction of each month. Thus, data from the future are used for a current time. In this section a fairer analysis is performed, which reflects the intended usage in practice better, where data up to month 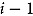 is used for predicting month 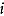. In performing this analysis, the model is given one initial year of data, after which repeatedly one month is added, and the next month predicted. The result is shown in Figure 6. The model starts with large confidence intervals, and inaccurate predictions. However, from 2013 onward the prediction looks acceptable.

## Discussion

Data were collected on several aspects that are thought to be important in PUUV epidemiology: mast, bank vole abundance, bank vole infection, and the resulting human case data.

In the mast data, a two-year peaked pattern was observed over the years, which is similar to what was observed by others.[10] Initially, also the mast produced by the American Oak was included. Interestingly, it also exhibited a two-year cycle, but with lower amplitude and in anti-phase with the oak and beach mast. For this reason, and because inclusion lessened the predictive power, the American oak was excluded from the study.

For purposes of early warning, it is advisable to monitor masting intensity. Indeed, the model predicts that the mast of a previous year is the determining factor for bank vole abundance. This beneficial effect of mast was observed before, and explained by increased winter survival and extended breeding of bank voles in the summer, e.g. [10]. Note that the dynamics of voles is such that the vole abundance of the previous year has no significant effect on the vole population of the current year, since numbers typically decline to very low numbers in the winter, and thus the population abundance is almost ‘reset’, to a level influenced by the mast.

In [5] vole abundance as a function of mast is also studied. In line with our results, strong correlation with mast was found, and no proof of year–month interactions. It is worth noticing that [17] found no relation of vole abundance with vole infection status, which was also not present in our study. However, it was also put forward that a lagged relation may exist. For future analysis, this is worthwhile to examine in our dataset.

In human cases a peaked pattern is visible: 2010, 2012 and 2014 were clearly years with a high number of human cases in the Netherlands. In [18] such a pattern is discussed for Belgium, where since 1990 cycles of two or three years existed, and since 2005 no cycles have been observed. Those authors doubt the relation between masting and vole populations for recent years. The same article states that in France human cases followed a three-year cycle from 1990 to 2000. Most of the cases were reported in the summer, which mimics the Dutch situation. Interestingly, human cases in Belgium and Germany did not always peak in the summer months. In considering the human case data, it should be noted that a large proportion of PUUV infections are asymptomatic and therefore not recognised, and under-diagnosis of symptomatic PUUV infections in the Netherlands has been reported.[15]

Another modelling approach used more advanced dynamic regression models.[19] In agreement with our findings, they also found temperature and bank vole trapping indices predictive for human cases. The model also differs in the use of a lag in the effect of the covariates, which may be more difficult in our case, given the sparse data on trappings.

In [20] a model relating human disease to beech fructification and vole abundance is presented for several German federal states. The similarities in the collected data are striking. Concerning vole abundance, the same pattern was observed for all years except 2010 where a high abundance is recorded, while our data show both low (June) and high bank vole abundance (September). Also beech masting is comparable, with high mast production in 2009 and 2011, and low mast production in 2008 and 2010. Only for 2007 our data shows a different masting intensity. In concordance with our model, these authors also find good correspondence between mast, rodent abundance and human illness. The resolution however is on a yearly scale, as compared to a monthly scale in our model. Also the model is not used for predictions, and is not tested retrospectively for predictive performance.

It is known that weather conditions influence seed production of trees.[21] Thus, it would in principle be possible to build a model relating mast volume to climatic conditions. This would open the way to forecasting PUUV outbreaks using primarily climatic data, which is monitored and forecasted routinely. Thus, for future research, it would be interesting to study the relation between climatic variables and mast production, as attempted before.[9] A direct link between climate and human incidence was also studied,[10] and it was found that high temperatures in summer and autumn, one and two years before an outbreak were significant predictors.

It should be noted that the driving mechanisms behind bank vole population dynamics differ between temperate Europe and Fennoscandia. While in temperate Europe mast production is of most importance, it is predator–prey cycles which determine the dynamics in northern European climates.[11] Hence, the current study can only be considered to be representative for temperate European climates. Nonetheless, several features of the boreal setting seem also to occur in the temperate European regions. For example, in [4] it was found that human nephropathia epidemica disease case dynamics were synchronised with bank vole abundance. Furthermore, the precise dynamics of infection in the vole population was not required for accurate prediction of human cases, which is what we also observe.

In determining bank vole seropositivity, juveniles may be falsely scored positive due to influence of maternal antibodies.[22] In our data, about 10% could be identified as juveniles (and for one third the age class was not recorded). Potentially, maternal antibodies could therefore have influenced the results. Unfortunately, our data do not allow us to correct for this effect. This does not invalidate the model results, since infection prevalence was found to be only weakly contributing to the human infection rate.

We found no correlation between rodent infection prevalence and abundance, which is likely due to the sparsity of the data collected. Previous studies in Northern Europe did underscore the strong link between rodent population dynamics and prevalence. High prevalence was linked to high abundances of voles, with maternal antibodies having a dampening effect at high prevalence phases. In Belgium it was also observed that vole infection prevalences are coupled to abundance, i.e. density dependent transmission.[23] This could be a possible explanation for the phenomenon that vole infection prevalences often do not seem to be crucial in modelling human cases from vole abundances, since prevalence correlates strongly to abundance and is indistinguishable for a model based on limited data.

In summary, models for relating vole abundance to masting in the previous year were successfully established. Furthermore, the model is able to forecast human cases, using temperature data, and abundance of positive voles, or temperature and mast scores of the previous year. The model thereby contains a hazard component (abundance of positive voles, or mast), and a component that may be interpreted as a human exposure component (temperature). But, this cannot be established with certainty, because temperature may also influence e.g. virus survival in the environment.[1] The mast and vole abundances determine to a large extent the year-to-year variation, while the temperature determines mostly the within-year variation.
